# Collectively encoding protein properties enriches protein language models

**DOI:** 10.1186/s12859-022-05031-z

**Published:** 2022-11-08

**Authors:** Jingmin An, Xiaogang Weng

**Affiliations:** 1 School of Life Sciences, Northeast Agricultural University, Harbin, 150030 China; 2 State Key Laboratory of Membrane Biology, Institute of Zoology, Chinese Academy of Sciences, Beijing, 100101 China

**Keywords:** Protein language modeling, Multi-task learning, Transfer learning

## Abstract

Pre-trained natural language processing models on a large natural language corpus can naturally transfer learned knowledge to protein domains by fine-tuning specific in-domain tasks. However, few studies focused on enriching such protein language models by jointly learning protein properties from strongly-correlated protein tasks. Here we elaborately designed a multi-task learning (MTL) architecture, aiming to decipher implicit structural and evolutionary information from three sequence-level classification tasks for protein family, superfamily and fold. Considering the co-existing contextual relevance between human words and protein language, we employed BERT, pre-trained on a large natural language corpus, as our backbone to handle protein sequences. More importantly, the encoded knowledge obtained in the MTL stage can be well transferred to more fine-grained downstream tasks of TAPE. Experiments on structure- or evolution-related applications demonstrate that our approach outperforms many state-of-the-art Transformer-based protein models, especially in remote homology detection.

**Supplementary Information:**

The online version contains supplementary material available at 10.1186/s12859-022-05031-z.

## Introduction

Natural language is inherently context-dependent. This fact becomes particularly prominent when two strongly-related words are far separated. From a linguistics perspective, context plays a significant role in deciphering the actual meaning of a word [1]. Likewise, correctly encoding contextual information is essential for natural language processing (NLP). Much prior research employed deep learning methods, such as convolutional neural network (CNN) [2], Recurrent Neural Network (RNN) [3] and word embedding [4], to acquire such inter-word dependencies. Recent advanced attention-based models possess equally powerful representation abilities to capture these contextual relationships through the self-attention mechanism [5]. Similar to natural language, protein sequences also hold strong contextual information, implicitly denoting structural, evolutionary or functional characteristics [5]. Appropriately capturing these inter-residue relationships from the sequence is of great interest to computational biologists. Considering the co-existing contextual relevance between natural and protein language, a sophisticated NLP model can likewise learn contexts in protein language. Many researchers have studied this aspect, and BERT [6] is one of the most popular NLP architectures. These studies can be classified into two types, depending on whether they introduce protein knowledge in the pre-training stage. The first type aims to construct a protein language model by pre-training on a large protein corpus [5, 7–11]. The other type directly transfers knowledge in human words to decode protein language [12–14], demonstrating slightly poor performance compared to that of the models pre-trained on protein language. Indeed, the pre-training stage is imperative for improving results on downstream tasks [8]. However, it is still unclear whether the performance of costly pre-training on a large in-domain corpus certainly will outperform that when transferring learned knowledge from natural language into domain-specific tasks. In addition, most second-type research simply transfers natural language embeddings to learn protein representations by fine-tuning specific tasks without following protein in-domain re-training. Admittedly, the abundant contextual representations encoded by human language models can naturally be used to capture such context in proteins. Most importantly, delicately enriching protein knowledge by in-domain protein tasks is greatly helpful for deciphering useful protein properties. Therefore, with the help of BERT pre-trained on large natural language corpus, together with encoding protein properties from closely-related protein tasks, such protein language models is expected to get promising downstream results.

Multi-task learning (MTL), which is able to leverage useful information of related tasks to achieve simultaneous strong performance on multiple associated tasks [15], has led to great success in many machine learning applications like NLP [15, 16]. As for the protein sequence domain, MTL has been widely applied for functional studies, like protein–protein interaction and protein targets [17–20]. A notable work [21] fused self-supervised language modeling and four supervised tasks in a model, realizing an end-to-end MTL architecture. Specifically, they employed two residue-level (secondary structure prediction in 3- and 8-states) and two protein-level (subcellular localization prediction and the classification membrane-vs-soluble proteins) tasks, which enables the model to jointly decipher protein properties and transfer knowledge between these different tasks. However, the multiple supervised tasks they adopted are not highly dependent, and they did not test the model performance on downstream tasks either. There are many correlated tasks in the protein domain, such as structural similarity and contact prediction, contact prediction and remote homology detection. However, little research focused on the interrelated protein tasks to facilitate the survey of protein structure or evolution. It is worth mentioning that Charuvaka et al. [22] employed every hierarchical category from the Structural Classification of Proteins (SCOP) [23] and CATH [24] databases as a single classification task to predict the structural type of protein sequences. SCOP [25] is a popular protein database that hierarchically classifies protein domains into four categories, listed from the bottom to the top: family (the proteins that share the exact evolutionary origin), superfamily (the proteins that evolved from the same ancestor but are distantly related), fold (the proteins that hold the same global structural features), class (the proteins gathered from fold and superfamily that have specific secondary structural content). To be precise, family explicitly denotes the evolutionary relations between proteins while superfamily gathers proteins with similar structure but less sequence similarity. Fold groups superfamilies based on the global structural features shared by most of their members and the constituted families can evolve distinct structures. Accordingly, we can clearly realize the intrinsically-related evolutionary and structural properties among family, superfamily and fold categories. Villegas-Morcillo et al. [26] adopted pairwise fold recognition (PFR) and direct fold recognition (DFC) tasks to identify protein fold category. However, they only focused on the classification performance toward fold label without collectively employing abundant information behind these three labels. Comprehensively considering the information behind the three categories is expected to encode important evolutionary and structure in prior knowledge, which could further be transferred to related downstream tasks. In this paper, therefore, based on the three closely-related classification tasks, we designed a MTL architecture to capture such structural and evolutionary relationships.

Transfer learning means transferring the knowledge from a related task that has already been learned to a new task [27]. Rives et al. [9] pointed out that learning intrinsic biological properties directly from protein sequences can further be transferred to prediction and generation. Likewise, Bepler et al. [5] showed that transfer learning could potentially improve downstream applications in certain scenarios. Through learning two supervised structural tasks, they found that the performance of their protein language model on two function tasks had been improved. It is worth noticing that these learning procedures are based entirely on protein sequences. Different from their work, we both introduced in prior knowledge of the natural language and protein sequences in the pre-training and multi-task learning stages, respectively. Another noteworthy work [28] used three types of fine-tuning protein tasks, including sequence classification, sequence-pair classification and token-level classification, ultimately improving several downstream performances and demonstrating the effectiveness of transfer learning for protein in-domain tasks. Tasks assessing protein embeddings (TAPE) [8] provides standardized benchmarks to evaluate the performance of learned protein embeddings. It contains five biologically-relevant tasks with regard to structure prediction, evolutionary understanding and protein engineering domains. Among these benchmark tests, we chose secondary structure prediction, contact prediction and remote homology detection as the downstream tasks to verify the transfer ability of our MTL models.

**Fig. 1:**
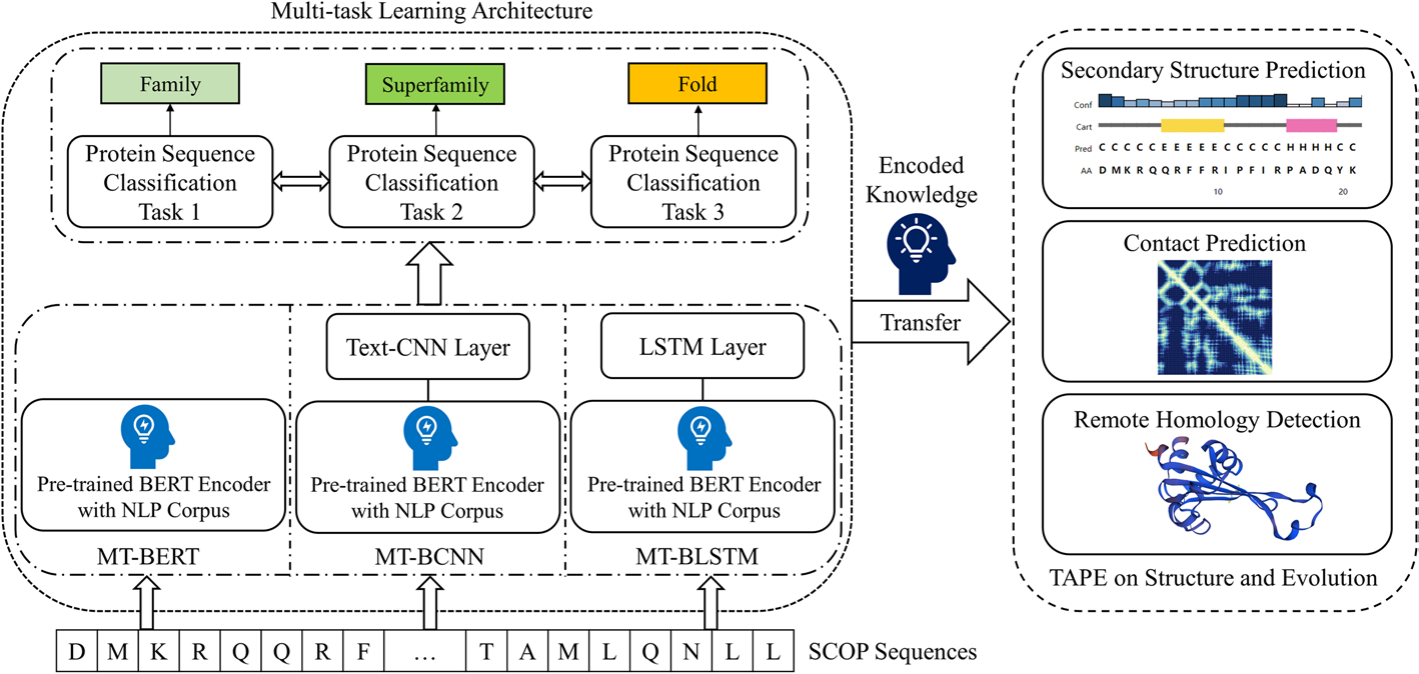
Workflow of our method. The colored rectangles represent three interrelated protein labels defined in SCOP 2 [25]. The predicted secondary structure sequence, contact map and 3D structure are illustrated by PSIPRED [29], ProteinTools [30] and Swiss-model [31], respectively (PDBid: 3H8D)

In this work, to sum up, we proposed a multi-task learning framework using three BERT-based backbones, which employed abundant contextual representations obtained in natural language and jointly learned knowledge on interrelated protein tasks. Three structural- or evolutionary-relevant downstream tasks, well-defined in TAPE, were used to evaluate whether our MTL architectures properly capture the structural and evolutional relationships. The overall workflow is shown in Fig. 1.

## Materials and methods

Overall, we elaborately designed three MTL backbones with different intrinsic architectures, namely MT-BERT, MT-BCNN, and MT-BLSTM. To jointly learn protein structure and evolution properties, we assigned these models a multi-task classification pipeline with respect to protein family, superfamily and fold categories. Finally, the learned knowledge was transferred to decode fine-grained applications well-defined in TAPE. Specifically, we adopted three structural- or evolutionary-related downstream tasks, including secondary structure prediction, contact prediction, and remote homology detection, to evaluate the transfer learning performance of our proposed MTL models.

### MTL models

#### MTL datasets

The training and test datasets we used for the MTL pipeline are all derived from SCOP 2 [25], a widely-applied database that aims to encode structural and evolutionary relationships between proteins. We first extracted the label information directly downloaded from this database, and then located the corresponding sequence as per the superfamily-level domain identification. Until May 1st 2022, the SCOP 2 dataset provides 36,534 well-labeled amino acid sequences. Moreover, to avoid information leakage in the MTL training stage and downstream test phase, we eliminated 367 overlapped sequences between TAPE remote homology detection test set and our whole MTL dataset. After that, we split the cleaned dataset (a total number of 36,167 sequences) into training and test sets on a scale of 7:3. The statistics [25] of our reconstructed dataset for these labels are in Table 1, and we can see that a great many types are included in each label. Additionally, the number of protein sequences for each type is quite unbalanced (e.g., only several proteins are classified into a specific type). Therefore, we ensured that every type in each label includes at least one training sequence to avoid a test protein belonging to an unknown type.

**Table 1:** Statistics of family, superfamily and fold categories in our MTL dataset

Statistics	Family	Superfamily	Fold
Number	5842	2750	1577

#### MTL backbones

For all NLP models, pre-training on a large natural language corpus is essential for learning universal language representations [32]. As a pioneering and representative work, BERT [6], a variance of Transformer [33], demonstrates powerful transfer learning ability with pre-training bidirectional representations from amounts of unlabeled natural language data. It has been shown that the prior natural language knowledge encoded in NLP models can be well transferred to handle biological sequences [12–14]. Additionally, BERT attention captures the folding structure of proteins, targets binding sites and focuses on progressively more complex biophysical properties with increasing layer depth [34]. Accordingly, we employed BERT [35] pre-trained on 3300 M human words as part of our MTL backbones, and the one that did not follow any other sequence analysis networks acts as our MT-BERT architecture.

The powerful feature extraction ability of BERT with substantial parameters may overfit the classification task, especially with limited training data [36]. CNN has demonstrated its great potential in the application of image data [37]. Besides, it has achieved equally strong performance on text classification [2] and natural language modeling [38], even though it is not as frequently used as in images. In such a structure, neurons between different layers are partially connected, which can well reduce intrinsic noises in protein sequences that hinder language models from deciphering protein properties. Therefore, introducing CNN to BERT can avoid overfitting to a certain extent. Additionally, Long Short-Term Memory (LSTM) [39], a variant of RNN, first came out to address the difficulty of storing long-range sequence information. The fact of linearly encoding of input sequence enables LSTM to retain relative intra-sequence dependencies better. Accordingly, LSTM is especially suitable for encoding distantly-related and order-depended structural and evolutionary relevance in protein sequences. Therefore, based on the above considerations, we respectively added CNN and LSTM layers to the final BERT encoder, becoming the backbones of the so-called MT-BCNN and MT-BLSTM.

Compared to single-task learning, which learns only one specific representation for once, multi-task learning enables the knowledge learned in one task to benefit other tasks [40]. As mentioned in “Introduction” section, there is a strong correlation among the pre-labeled protein family, superfamily and fold categories. Hence, we adopted three classification tasks for these labels to enable our MTL models to encode implicit evolutionary and structural information.

#### Details of MTL architecture

**Fig. 2:**
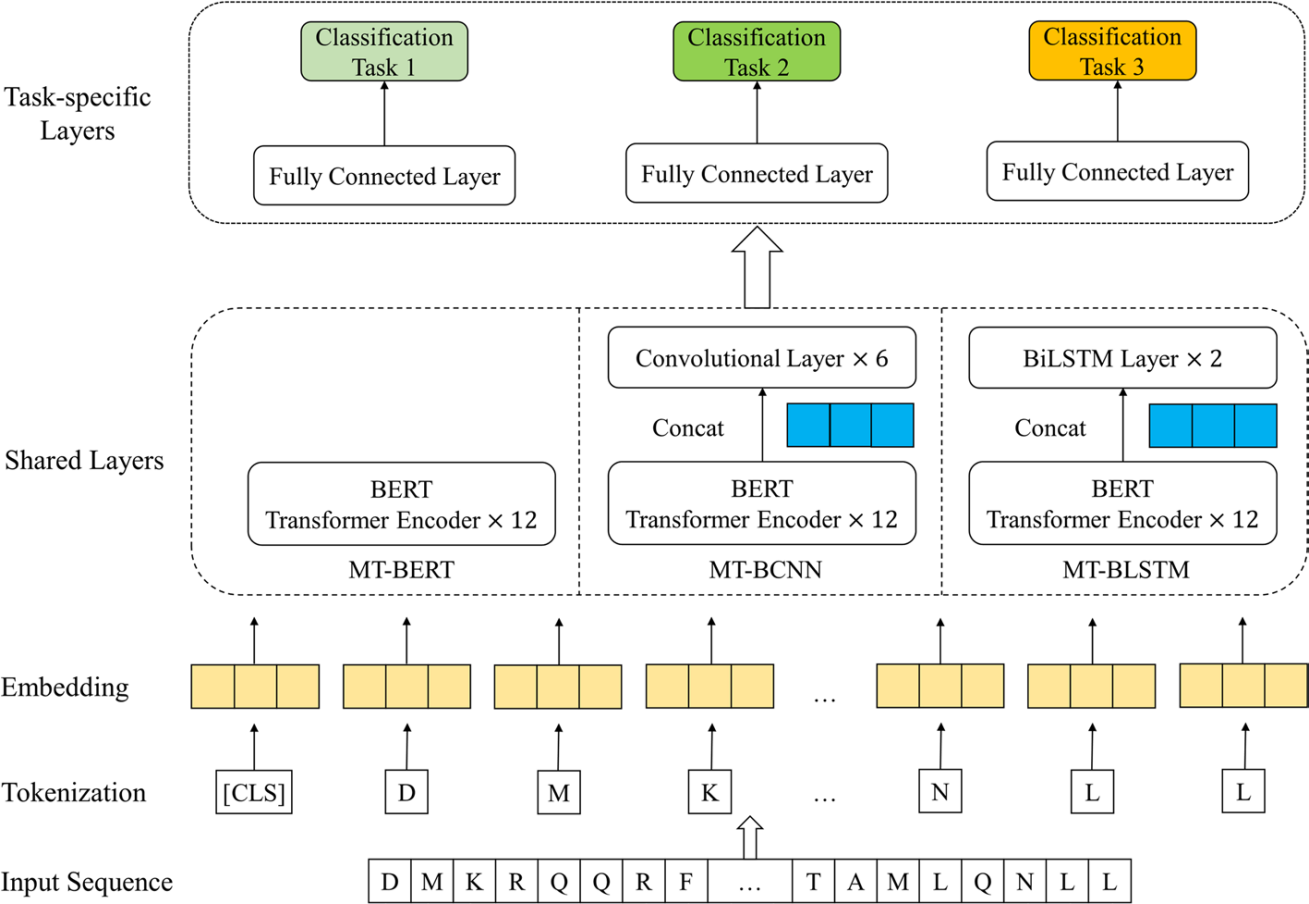
The detailed structure of the proposed MTL framework with three kinds of backbones

Deep learning, which is inherently specialized in learning complex non-linear feature representations [41, 42], has been widely applied in MTL domains. Zhang et al. [42] classified deep MTL models into two main types: learning common feature mappings by sharing the first several layers or introducing adversarial learning; learning different feature mappings with a cross-stitch network. They also pointed out that only sharing hidden layers is quite powerful when all the tasks are correlated. Since we defined three closely-related classification tasks of the protein family, superfamily and fold, our MTL models also share previous layers to learn common structural and evolutionary representations. Inspired by Liu et al. [40], we proposed an improved deep MTL architecture specially designed for modeling protein language (see Fig. 2).

Every input protein sequence would first be tokenized into separate amino acids represented by specific alphabets, then embedded in a maximum of 8096 vector spaces according to the length of the sequence. Note that the $[CLS]$ token is used for sequence-level classification tasks. After that, these embeddings would all go through BERT Transformer Encoder layers for extracting contextual information. The difference between our three MTL models lies in the shared layers. MT-BCNN and MT-BLSTM concat the context embeddings from the last BERT Encoder with six CNN layers and two bidirectional LSTM layers, respectively. The output representations of the shared layers would finally feed into three task-specific fully connected layers for classifying protein family, superfamily and fold.

Generally, an MTL model can be trained by linearly combining loss functions from different tasks into a single total loss function [15]. In this way, the model can learn a shared representation for all tasks by stochastic gradient descent (SGD) with back-propagation [15, 43]. Ordinarily, assuming that there are $M$ tasks in all, the global loss function can be defined as

1 $$\begin{array}{*{20}c} {L_{total} = \mathop \sum \limits_{i}^{M} w_{i} L_{i} } \\ \end{array}$$

where ${L}_{i}$ represents task-specific loss function, and ${w}_{i}$ denotes weights assigned for each ${L}_{i}$.

It is worth noticing that the performance of MTL models strongly depends on the relative weighting between the loss of each task [44]. It has been reported that many researchers set these weights according to experience or through costly grid search [15]. Following the previous work of Kendall et al. [44], we adopted homoscedastic tasks uncertainty to optimize the loss weights ${w}_{i}$. Moreover, we used cross-entropy loss function for each classification task:

2 $$\begin{array}{*{20}c} {L_{i} = - \mathop \sum \limits_{c} I_{d} \left( {X,c} \right)\log p\left( {X{|}c} \right)} \\ \end{array}$$

where $X$ denotes the input protein sequence, ${I}_{d}(X,c)$ is a binary identification (0 or 1) indicating whether the label $c$ is the correct category of $X$, and $p(X|c)$ represents the predicted probability that $X$ is classified to label $c$*.*

Here we demonstrate how to train our MTL models in Algorithm 1. The same MTL dataset, derived from SCOP 2, was used to jointly learn how to classify proteins into family, superfamily and fold. We set a batch size of 32, a dropout rate of 0.1 for BERT and 0.4 for LSTM. We defined a larger learning rate of 1e-2 for CNN and LSTM connected after BERT, while the pre-trained BERT held a relatively small learning rate of 1e-5. All the above hyperparameters were fine-tuned through Bayesian Optimization [45]. We also employed SGD to update model parameters step by step. The training procedure was implemented with PyTorch [46] on NVIDIA Quadro GP100. 

#### Dealing with long protein sequences

Among the three proposed MTL models, the protein sequences are always first fed into BERT Encoder (see Figs. 1, 2). The maximum input of the pre-trained BERT model on natural language is set to 512, while the length of amino acid sequences can sometimes exceed it. Sun et al. [32] proposed three ways to deal with long natural language articles: head-only, tail-only and head–tail. However, these novel solutions cannot readily handle protein sequences since every residue may represent unique structural and evolutionary information. Thus, instead of cutting up residues, we must keep the whole sequence as the input for the following embedding process. We first re-initialized the length of the max positional embedding dictionary to 8096, the same size as that in TAPE [8]. Then we replaced the randomly initialized first 512 tokens in the whole 8096 tokens with the previously-encoded position embeddings in pre-trained BERT. In doing so, we not only retain the encoded representations obtained in natural language pre-training, but can further embed the rest 7584 vectors in the following MTL and downstream protein tasks.

#### MTL model evaluation metrics

The performance of our models in the MTL stage can not only partly influence that on downstream scenarios, but also validate whether abundant natural language knowledge is well transferred to encode protein properties. Therefore, we report four evaluation metrics to estimate the sequence-level classification performance: Precision (Pre), Accuracy (Acc), Recall (Rec) and F1-score (F1). These indexes are frequently used to assess the generalization of machine learning models from distinct perspectives. The detailed definitions can be seen below.

3 $$\begin{array}{*{20}c} {Precision = \frac{TP}{{TP + FP}}} \\ \end{array}$$

4 $$\begin{array}{*{20}c} {Accuracy = \frac{TP}{{TP + TN + FP + FN}}} \\ \end{array}$$

5 $$\begin{array}{*{20}c} {Recall = \frac{TP}{{TP + FN}}} \\ \end{array}$$

6 $$\begin{array}{*{20}c} {F1 - score = \frac{2*Precision*Recall}{{Precision + Recall}}} \\ \end{array}$$

### Downstream tasks

MTL aims to help improve the generalization of all tasks [42]. In this study, however, we want to investigate how the jointly-learned protein knowledge could facilitate relevant downstream tasks. In other words, we would like to see whether the encoded structural and evolutionary information can transfer to decode more fine-grained assignments. To test the transfer ability of our MTL models, we therefore employ two structure prediction and one evolutionary understanding tasks in TAPE [8]. Furthermore, all datasets and metrics used to evaluate our models are identical to those in TAPE to ensure comparability.

#### Secondary structure prediction

**Fig. 3:**
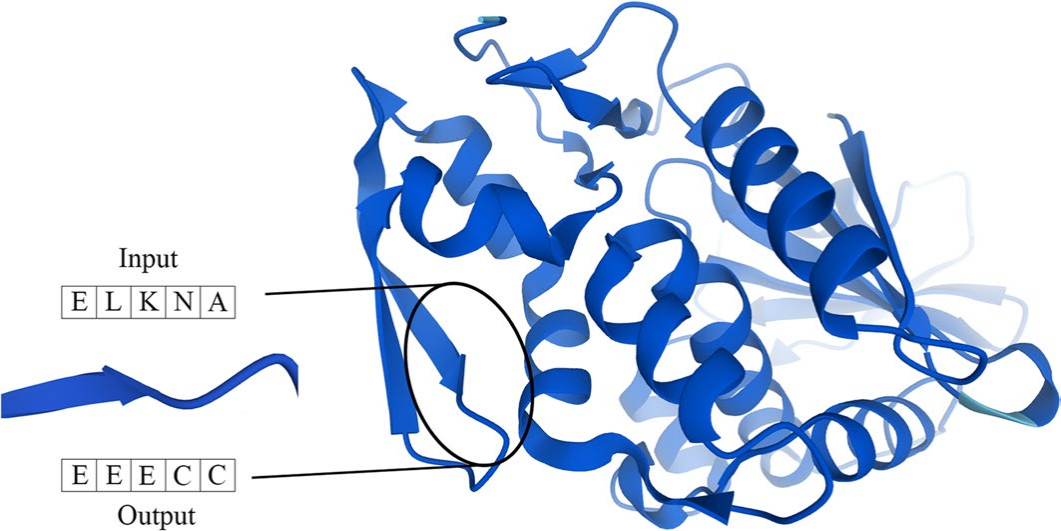
Illustration of secondary structure prediction, where the 3D structure is established by Alphafold [52] (PDB id: 1J1Q)

Secondary structure prediction (SS prediction) is a sequence-to-sequence classification assignment. Assuming that there is a protein sequence, this task is dedicated to labelling every input amino acid with a secondary structure position (see Fig. 3). The labels can further be categorized into 3-state secondary structure (i.e., alpha-helix (H), beta-strand (E) and coil region (C)) and 8-state secondary structure (i.e., helix (G), α-helix (H), π-helix (I), β-stand (E), bridge (B), turn (T), bend (S), and others (C)) [47–49]. We often evaluate the performance of SS prediction by Q3 or Q8 accuracy, which measures how many residues for which 3-state or 8-state secondary structure is correctly predicted [50]. Accurate SS prediction facilitates the study of protein structure and function [47], including fold-recognition, homology modeling, ab initio and constraint-based tertiary structure prediction, as well as identification of functional domains [51].

As in TAPE, the training and validation datasets for secondary structure prediction are from [53], and Q3 accuracy is reported on the test set CB513 [54].

#### Contact prediction

**Fig. 4:**
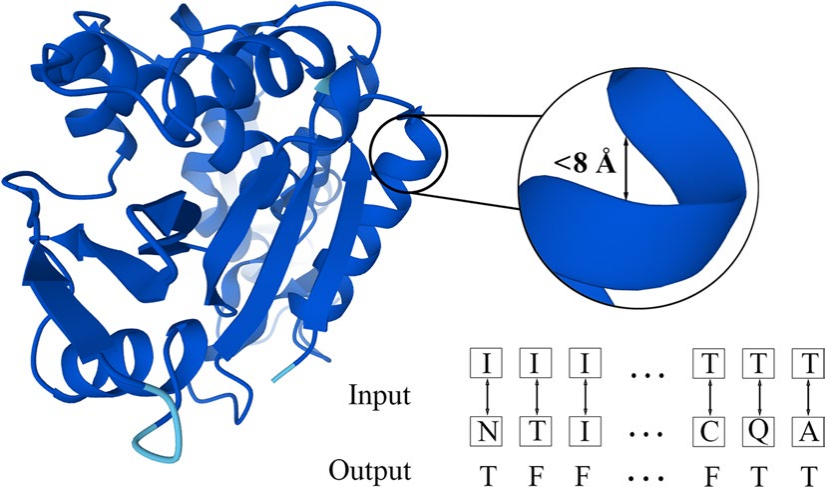
Illustration of contact prediction, where the 3D structure is established by Alphafold [52] (PDB id: 1J1Q)

Contact prediction is a pairwise classification assignment. Given a protein sequence, the goal of this task is to predict whether each pair of residues from this sequence are “in contact” (typically, it is defined as the distance in folded structure < 8 Å [8, 49]) or not (see Fig. 4). The contacts can be subdivided into short-, medium- and long- ranges corresponding to the sequence separation equal to 6–11, 12–24 and > 24 respectively [55]. Correctly-predicted contacts capture powerful global structural and folding information [8, 56], facilitating 3D structure modeling, especially de novo protein structure prediction [57].

As in TAPE, the dataset for contact prediction is from ProteinNet [58]. The precision of the $L/5$ most likely contacts for medium- and long-range contacts, where $L$ is the length of protein sequence, are reported on the ProteinNet CASP 12 test set [59].

#### Remote homology detection

**Fig. 5:**
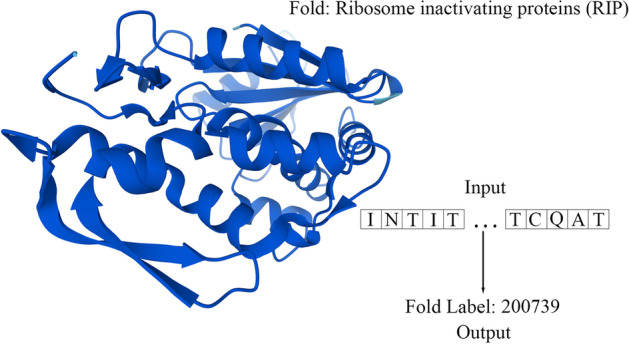
Illustration of remote homology detection, where the 3D structure is established by Alphafold [52] (PDB id: 1J1Q)

Remote homology detection is a conventional sequence-level classification assignment. Since distantly related proteins may share similar structures and functions [60], this task targets to predict which fold structure the input protein sequence belongs to (see Fig. 5). This fold structure is the exact fold label clearly defined in SCOP [25]. Protein remote homology is critical for studying protein structures and functions [55] and drug design [61]. It identifies proteins from different families and therefore is suitable for predicting the structure and functions of specific proteins [62]. Note that this assignment is similar to the fold classification task implemented in the MTL stage, and significantly improved performance is reported compared to other SOTA work in  “Result” section.

As in TAPE, the dataset of remote homology detection comes from [63], which originates from SCOP 1.75 database and Protein Data Bank [64, 65]. The overall classification accuracy is reported on the fold-level heldout test set from [63]. It is worth mentioning that the dataset we used in the MTL stage derives from SCOP 2 (a successor to SCOP 1.75); thus, the test set in TAPE may contaminate our training set. Therefore, we screened the overlapped proteins in our constructed MTL dataset for accuracy concerns.

## Results

### MTL model evaluation

Using the four evaluation metrics reported in  “MTL model evaluation metrics” section, we first estimated the model performance on family, superfamily and fold classification tasks in the MTL phase. The reported results are averaged over the three classifications based on tenfold cross-validation (see Table 2).

**Table 2:** Averaged results of the tenfold cross-validation on our MTL training set

Model	Pre	Acc	Rec	F1
MT-BERT	0.636	0.643	0.705	0.662
MT-BCNN	0.705	0.767	0.742	0.723
MT-BLSTM	**0.731**	**0.771**	**0.756**	**0.743**

These results validate that the knowledge in natural language can indeed transfer to handle sequence-level protein classifications. Furthermore, compared to MT-BERT solely employed BERT, the introduced CNN and LSTM layers in MT-BCNN and MT-BLSTM have improved the overall classification performance. Specifically, we can see that MT-BLSTM gets the best results among all the three MTL models.

### The effectiveness of MTL

**Fig. 6:**
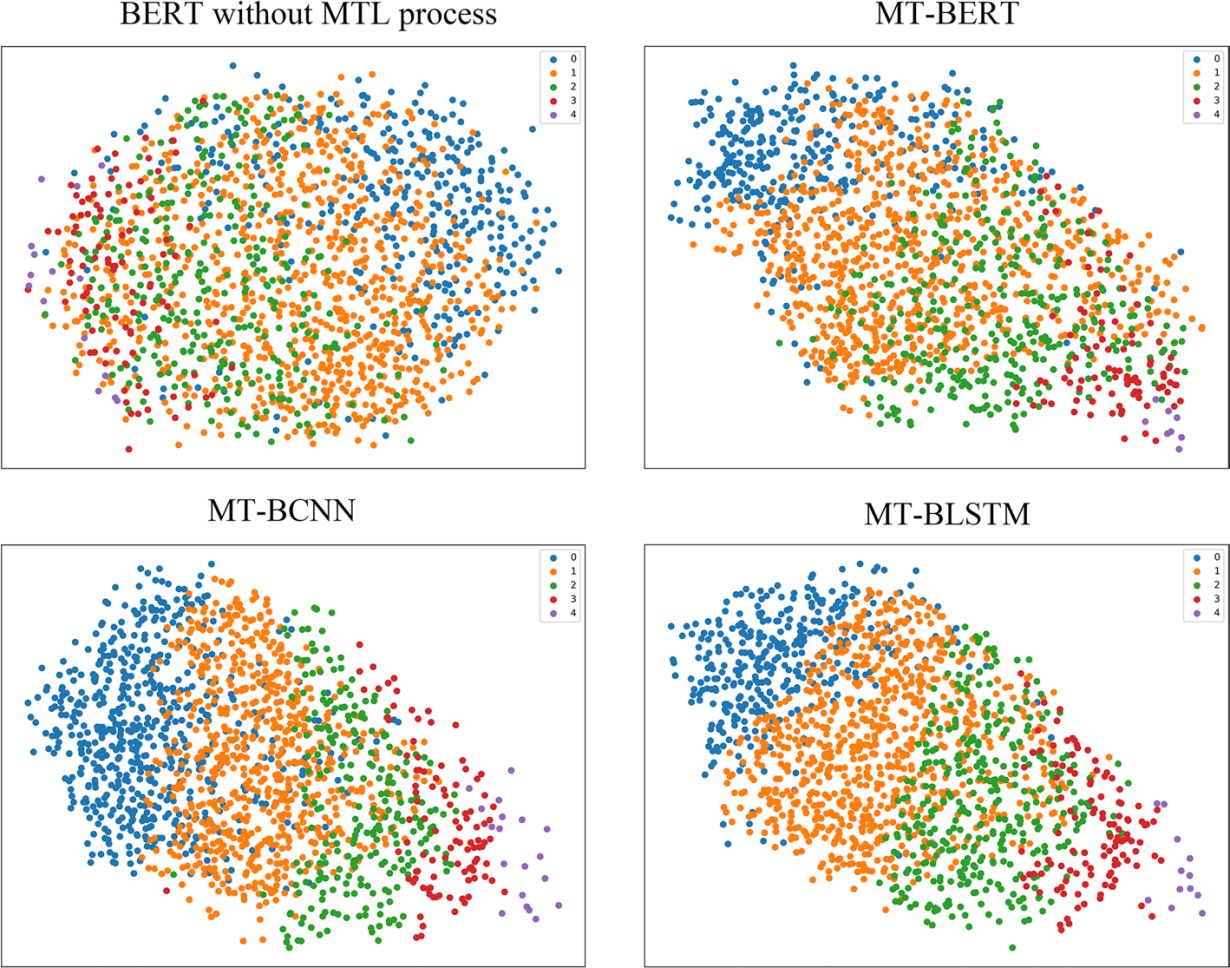
Comparison of manifold embedding of MTL dataset proteins. Different colours represent distinct labels in the Class Category

As mentioned above, we considered three of the four protein categories, namely family, superfamily and fold, and the information behind the class label has not been employed. Class category gathers folds and intrinsically-unstructured proteins from superfamily, which indicates a solid structural concept and the correlation with evolution. For this time, this label can be used to verify the effectiveness of our MTL models. We compared the learned features between the original pre-trained BERT and our MTL models to check whether these multiple tasks encode useful structural and evolutionary information. Generally, an MTL model would encode a given sequence into high-dimensional vector embeddings. However, it is possible to map the whole semantic space by pooling them into fixed-size embeddings by reduction [5]. Moreover, introducing clustering and manifold embedding to visualize large protein datasets can reveal structural and evolutionary relationships between sequences [5]. Thus, we compared the embedding results of pre-trained BERT without the MTL process with those of our MTL models. Figure 6 shows the visualized proteins in our whole MTL dataset after embedding and dimensionality reduction by Multidimensional Scaling [66–68]. The pre-trained BERT on natural language, without MTL protein-domain tasks, demonstrates inadequate structural classification ability, and the embedding spaces are significantly sparse and mixed. Three jointly-learned interrelated protein tasks are allocated, making the boundaries between distinct class labels clearer. Overall, it can be noticed from those embedded proteins that the MTL process improves the clustering performance. Furthermore, to statistically analyze the embedding differences of different models in Fig. 6, we evaluate the classification performance toward the class label on our whole MTL dataset. Table 3 reports detailed results using the same classification metrics as Table 2.

**Table 3:** Class label classification performance on our whole MTL dataset

Model	Pre	Acc	Rec	F1
BERT	0.232	0.186	0.219	0.225
MT-BERT	0.565	0.557	0.593	0.579
MT-BCNN	0.716	**0.736**	0.667	0.691
MT-BLSTM	**0.745**	0.726	**0.717**	**0.731**

We can see from Table 3 that the classification performance of our MTL models significantly outperforms the original BERT model that did not implement the MTL process, which is consistent with the manifold embeddings in Fig. 6.

Overall, such abstract clustering representations and statistical results proved that our MTL models captured useful structural and evolutionary information that could further facilitate related downstream tasks.

### MTL model performance on downstream tasks

We evaluated the MTL model performance on three downstream applications: secondary structure (SS) prediction, contact prediction and remote homology detection. Depending on the task, we reported the accuracy or precision described in “Downstream tasks” section as done in TAPE [8]. Each metric has a maximum value of 1.0, and higher represents the better. Note that the evaluation metrics remain the same in the following experiments.

Table 4 compares our three MTL models with the TAPE BERT [8], ProteinBERT [7], BERT medium [69], String2Seq [28] and ProtBert [11]. Except the secondary structure prediction dataset used in the BERT medium was from [70], all the results in Table 4 are reported on the same datasets described in “Downstream tasks” section. We can notice that MT-BLSTM and MT-BCNN obtained the best results under these three structural or evolutionary tasks. Notably, the performance of remote homology detection had been significantly improved compared with other SOTA models. This phenomenon can partly be attributed to the close relationship between fold label classification in the MTL stage and the essence of remote homology detection, both of which view fold category as a classification task. In conclusion, our MTL models effectively deciphered underlying protein properties and obtained well downstream performance.

**Table 4:** Comparison of TAPE benchmark results on three structure- or evolution-related tasks

Model	Structure		Evolution
	SS Prediction	Contact Prediction	Remote Homology
TAPE BERT	0.73	0.36	0.21
Protein- BERT	0.74	–	0.22
BERT medium	0.74	–	–
String2Seq	**–**	–	0.25
ProtBert	0.80	–	–
MT-BERT	0.75	0.39	0.32
MT-BCNN	**0.82**	0.43	0.39
MT-BLSTM	0.77	**0.45**	**0.42**

#### Comparison of pre-training on natural language and protein language

Compared to other Transformer-based models that acquire in prior knowledge of proteins, we can see that the BERT pre-trained on natural language in our MTL models can get good transfer learning performance. Furthermore, these results can verify the importance of introducing in-domain knowledge to natural language pre-training models. In other words, appropriately encoding protein property information can significantly boost the performance of downstream applications. More than that, it is essential to validate if pre-training on protein sequences outperforms pre-training on natural language corpus. To do this, we employed two BERT-based models TAPE BERT [8] and ProteinBERT [7], pre-trained on 31 M protein domains from Pfam [71] and ~ 106 M proteins from UniProtKB/UniRef90 [72] respectively. Moreover, the structure and parameters of TAPE BERT are almost identical to our MT-BERT, and the major difference between them lies in the pre-training corpus. Therefore, the performance of TAPE BERT can approximately reflect that of our MT-BERT if it is pre-trained on protein sequences. The ProteinBERT and TAPE BERT underwent the same MTL process as we did on our MTL models. Table 5 reports the downstream results on the same TAPE benchmarks.

**Table 5:** Comparison of TAPE benchmark results of protein sequences and natural language pre-training models after the MTL process

Model	Structure		Evolution
	SS prediction	Contact prediction	Remote homology
TAPE BERT(MT)	**0.79**	**0.42**	**0.32**
Protein-BERT(MT)	0.77	0.34	0.30
MT-BERT	0.75	0.39	**0.32**

TAPE BERT(MT) and ProteinBERT(MT) are used to distinguish the original ones that did not implement the MTL process

Compared to our basic model MT-BERT, we can see that pre-training on protein data significantly improves transferred performance. However, the best results are comparable with our MT-BCNN and MT-BLSTM models that rely on human words pre-training in Table 4. Furthermore, the increased results compared with the original TAPE BERT and ProteinBERT (see Tables 4 and 5) demonstrate the necessity of our MTL process for downstream tasks. In general, as Sun et al. [32] said, within-task and in-domain pre-training can largely boost the performance of BERT. However, the delicately-designed MTL models like MT-BCNN and MT-BLSTM can largely narrow the gap. In other words, pre-training on in-domain protein language deserves to perform better, but this is not the main point to be focused on. The MTL process indeed enriches protein properties, and the most predominant increase exists in remote homology detection. Therefore, the most important thing is how to subtly bring in strong biological priors, such as structure- or evolution-related information.

#### Ablation study employing two classification tasks

Moreover, exploring which two of the three tasks provide relative critical information is equally meaningful. Since the former three supervised tasks are closely related, we thereby tested how these well-designed MTL models perform if one of the tasks is missed (see Tables 6, 7, 8).

**Table 6:** Comparison of TAPE benchmark results based solely on family and superfamily classification task

Model	Structure		Evolution
	SS prediction	Contact prediction	Remote homology
MT-BERT	0.71	0.34	0.21
MT-BCNN	**0.75**	0.35	0.23
MT-BLSTM	0.72	**0.38**	**0.25**

**Table 7:** Comparison of TAPE benchmark results based solely on family and fold classification task

Model	Structure		Evolution
	SS prediction	Contact prediction	Remote homology
MT-BERT	0.73	0.33	0.28
MT-BCNN	**0.77**	**0.37**	0.32
MT-BLSTM	0.75	**0.37**	**0.35**

**Table 8:** Comparison of TAPE benchmark results based solely on fold and superfamily classification task

Model	Structure		Evolution
	SS prediction	Contact prediction	Remote homology
MT-BERT	0.73	0.35	0.29
MT-BCNN	0.74	0.39	0.34
MT-BLSTM	**0.75**	**0.41**	**0.37**

After removing one specific task, we can see an overall degraded performance with varying degrees. The combination of superfamily and fold tasks gets the best overall outcome in the ablation study. As described in SCOP 2 [25], the family and fold label explicitly denote the ancestor and space structure of proteins respectively, while the proteins in the superfamily usually share a similar structure. The results of this ablation study are basically consistent with the characteristics of proteins in different categories.

Overall, the learned representations by two related tasks can still be well transferred to downstream scenarios. However, the best results in these applications occur when all three highly-dependent classification tasks are considered.

#### Ablation study employing single classification task

Finally, to validate if the reduced complexity of single-task learning could influence the model performance, we solely adopt one of the three classification tasks to enrich our models. Note that ST-BERT, ST-BCNN and ST-BLSTM denote a single-task learning version compared with three MTL models, in which the whole model architectures remain the same.

Table 9 shows the model performance based on single-task learning. Overall, these results are not competitive enough compared to those of MTL models when multiple tasks are involved. Notably, the single fold classification task significantly improved the performance of remote homology detection. Moreover, this task also enabled the ST-BCNN model to obtain the best contact prediction result. Additionally, the superfamily category information may better specialize in predicting secondary structure.

**Table 9:** Comparison of TAPE benchmark results based solely on one classification task

Model	Task: family			Task: superfamily			Task: fold		
	SS	Contact	Remote	SS	Contact	Remote	SS	Contact	Remote
ST-BERT	0.68	0.30	0.15	0.71	0.29	0.14	0.69	0.28	0.20
ST-BCNN	0.72	0.32	0.17	**0.73**	0.29	0.18	0.70	**0.33**	0.23
ST-BLSTM	0.70	0.31	0.18	0.71	0.28	0.20	0.72	0.30	**0.26**

SS, Contact and Remote denote SS prediction, contact prediction and remote homology detection, respectively

## Discussion

The advanced NLP models, pre-trained on abundant natural language corpus, can be well transferred to decode biological sequences. Combined with the supervised training on multiple interrelated in-domain tasks, we demonstrate that these powerful NLP models can even outperform those fully modeling on protein language. Additionally, our approach further validates that transfer learning indeed improves downstream applications [5]. Furthermore, it enlightens us that costly pre-training on in-domain language corpus may not be indispensable, since our MTL models transferred knowledge from natural language and obtained competitive results in protein tasks (see “Comparison of pre-training on natural language and protein language” section). Conversely, the most fundamental part lies in how the in-domain knowledge can be subtly introduced. On the one hand, pre-training on a large natural language corpus enriches advanced NLP models abundant in prior knowledge, which can be well utilized to transfer to other domains. On the other hand, the way of in-domain re-training plays a leading role in improving model performance. It is generally accepted that jointly learning interrelated tasks can leverage important information, thus outperforming sing-task learning. [15]. Considering the many interrelated tasks in the protein domain, we can then comprehensively employ these tasks together. In this study, we adopted three classification tasks towards family, superfamily and fold categories hierarchically classified in SCOP 2, in order to encode implicit structural and evolutionary information from protein sequences.

Furthermore, we elaborately designed an MTL architecture. It contains three kinds of backbones: MT-BERT, MT-BCNN and MT-BLSTM. MT-BERT simply employs pre-trained BERT, while MT-BCNN and MT-BLSTM added CNN or LSTM layers to the top of BERT, aiming to avoid overfitting or better capture sequential invariance. Adequate experiments show that these models capture proper structural and evolutionary relationships by collectively learning from three correlated sequence-level classifications. Besides, the most critical part depends on the transfer learning ability. Among three challenging structure- or evolution-related tasks, the performance on remote homology detection has been significantly improved compared to other SOTA Transformer-based Models. Moreover, we can see the effectiveness of added CNN and LSTM layers in MT-BCNN and MT-BLSTM, which obtained better performance than MT-BERT.

Overall, we believe that our proposed methodology can facilitate the study of how to draw on sophisticated tools in natural language to learn protein language, as well as the way to encode strong biological priors into protein language models [5]. Further research can be focused on the MTL architecture itself. Since protein sequences differ from human sentences in structure and grammar, the most powerful MTL approach in NLP may not be the best protein language encoder. Moreover, employing other strongly-correlated tasks involving more fine-grained protein properties is expected to obtain promising downstream results as well.

## Supplementary Information

**Additional file 1**. Constructed protein dataset derived and cleaned from SCOP 2. Except for the protein sequence, each row of data contains specific SCOP domain identifications including FA-DOMID, SF-DOMID, CL, CF, SF, and FA, whose descriptions can be found at https://scop.mrc-lmb.cam.ac.uk/download.

## Data Availability

Constructed protein dataset derived and cleaned from SCOP 2 is included into the Additional file 1. Except for the protein sequence, each row of data contains specific SCOP domain identifications including FA-DOMID, SF-DOMID, CL, CF, SF, and FA, whose descriptions can be found at https://scop.mrc-lmb.cam.ac.uk/download.
